# Editorial: Plasticity in the Vertebrate Pituitary, Including Regulatory Mechanisms

**DOI:** 10.3389/fendo.2021.698916

**Published:** 2021-05-18

**Authors:** Romain Fontaine, Karine Rizzoti, Finn-Arne Weltzien

**Affiliations:** ^1^ Physiology Unit, Faculty of Veterinary Medicine, Norwegian University of Life Sciences, Ås, Norway; ^2^ Laboratory of Stem Cell Biology and Developmental Genetics, The Francis Crick Institute, London, United Kingdom

**Keywords:** pituitary, plasticity, adenohypophysis, hormones, stem cells, proliferation, transdifferentiation, regulation

## Introduction

In all vertebrates, the pituitary is involved in the control of many important and complex physiological processes such as growth, metabolism, homeostasis, reproduction, metamorphosis, and stress response. Located below the hypothalamus, the pituitary is divided into two main parts: the neurohypophysis (pars nervosa) and the adenohypophysis (pars distalis and pars intermedia). The latter, also named anterior pituitary (AP), is composed of up to 8 different hormone-producing cell types, highly specialized as they each mainly produce one type of hormone (1).

The adenohypophysis is characterized by its high plasticity, which is required to adapt hormone production to demand according to the environmental experience and life cycle of the animal. Pituitary plasticity occurs at three different levels (1): i) At the cellular level, cells can change activity in response to different signals by regulating receptor expression and by controlling hormone production and release; ii) At the structural level, cells can migrate and send projections that allows formation of homotypic and heterotypic networks that may influence the secretory behavior of the cells; iii) and finally at the population level, as endocrine cell numbers can be modulated. The latter encompasses differentiation of progenitor cells but also proliferation and changes of endocrine phenotype of specific hormone-producing cells which modify the cell type composition in the pituitary as a whole. Research questions such as when in the life cycle or under which environmental conditions does this plasticity play a role? What are the mechanisms allowing it? How are these mechanisms regulated? have been investigated for a long time but more intensely during the last decade due to the development of more powerful methodological tools.

On this basis, we conceived and coordinated the publication of a Research Topic on the “Plasticity in the Vertebrate Pituitary, Including Regulatory Mechanisms.” This e-book presents the collection of three original research articles and 8 reviews, covering the different mechanisms involved in the three levels of plasticity described above, the way they are regulated, and the role they play in the life cycle of an animal, across vertebrates.

## Mechanisms for Pituitary Plasticity

One original research article and five reviews discuss the different mechanisms involved in anterior pituitary plasticity.

First, an original research article from Rojo-Ruiz et al. explores the cellular level of plasticity by investigating the contribution of endoplasmatic reticulum (ER) calcium release following stimulation by two important hypothalamic signals (GnRH and TRH) in transgenic mice expressing an ubiquitous calcium sensor. The authors report that while GnRH and TRH induce robust decreases of ER calcium concentration and concomitant rises of cytosolic calcium concentration, depolarization with potassium triggers a rise of cytosolic calcium concentration alone, indicating that the calcium-induced calcium-release through ryanodine receptors is not present in AP cells. A mini review from Clay et al. also explores the plasticity at cellular level, focusing on gonadotropes. It discusses the functional and organizational plasticity, including changes in sensitivity to GnRH and in cellular morphology, and their critical role for the generation of the LH surge leading to ovulation.

Second, the mini review from Childs et al. discusses the presence and potential role of multihormonal cells and the functional plasticity in somatotropes and gonadotropes. The review from Laporte et al. extensively discusses the potential role for stem cells during physiological and pathological remodeling of pituitary endocrine populations, from maturation of the post-natal organ to tumoral development. Along with the mini review from Camilletti et al., it also provides an interesting overview of the major advances in the development of protocols to generate specific pituitary hormone-producing cell types from stem cells, and discuss their application in pituitary diseases.

Finally, the review from Santiago-Andres et al. focuses on the plasticity at the structural level and discusses the establishment and role of pituitary cell networks throughout vertebrate evolution. As most endocrine cell populations have been shown to form homo- and heterotypic networks in several vertebrate groups, this review highlights the way by which pituitary networks serve to optimize generation of hormone pulses in vertebrates.

## Regulation of Pituitary Plasticity

One original article and two reviews discuss the neuroendocrine and endocrine regulation of pituitary cells. Fleming et al. present an original research article on thyrotropes, characterizing the regulation of the two *tshb* genes that derived from the teleost-specific whole genome duplication (2) and are produced in two different cell types (3). Measuring their transcript levels in primary pituitary cell cultures, the authors report a differential regulation of the expression of the two *tshb* paralogs by CRH, somatostatin, TRH, thyroid hormones, cortisol and insulin-like growth factor 1. The review from Fontaine et al. focuses on the regulation of gonadotropes in fishes by internal factors, analyzing the extensive literature on the effect of sex steroids feedback mechanisms. It provides information on the complex effects of the different sex steroids which depend on their nature (androgens versus estrogens), on the species, sex of the animal and its physiological state. It discusses the three different levels of plasticity and discriminates direct effects on the gonadotropes from indirect effects at the brain and pituitary levels. In contrast, the second review from Vélez and Unniappan focuses on somatotropes and provides an overview and update on GH regulators, focusing on the main endogenous and synthetic factors, taking a comparative point of view within vertebrates.

One original article and one review discuss the environmental control of pituitary plasticity. The original research article from Ma et al. investigates seasonal changes in circulating LH and GH concentrations *in vivo* in female goldfish, and the sensitivity of transcript levels for several genes involved in reproduction and growth. The authors demonstrate the seasonality of LH, but not GH, circulating levels and the seasonally dependent effects of GnIH, GnRH and T3 on circulating levels of LH and GH. The authors also report seasonal patterns in basal and GnRH- and/or GnIH-induced transcript levels for ERα, ERβI, FSHR, aromatase, TRαI, TRβ, IGF-I, and VTG in the liver and ovary. The review by Ciani et al. summarizes the scarce literature on the regulation of anterior pituitary endocrine cell types by environmental factors through melatonin signaling in two vertebrate groups, mammals and fishes. It reveals the growing interest for the role of thyrotropes in the seasonal neuroendocrine regulation of the gonadotrope axis. Also distinguishing direct from indirect effects of melatonin, the comparison between these two vertebrate groups reveals similarities in several regulatory mechanisms.

In conclusion, this Research Topic offers an exhaustive update on the different aspects of pituitary plasticity, highlighting novels paradigms in primary research manuscripts, and synthetizing current knowledge in review articles. We hope that this compendium will remain useful to researchers interested in vertebrate neuroendocrinology.

## Author Contributions

RF wrote the manuscript. KR and F-AW edited the manuscript. All authors contributed to the article and approved the submitted version.

## Funding

This work was funded by the University of Life Sciences and in part by the Wellcome Trust [grant number FC001107].

## Conflict of Interest

The authors declare that the research was conducted in the absence of any commercial or financial relationships that could be construed as a potential conflict of interest.

## References

[1] Fontaine R Ciani E Haug TM Hodne K Ager-Wick E Baker DM. Gonadotrope Plasticity At Cellular, Population and Structural Levels: A Comparison Between Fishes and Mammals. Gen Comp Endocrinol (2020) 287:113344. 10.1016/j.ygcen.2019.113344 31794734

[2] Maugars G Dufour S Cohen-Tannoudji J Querat B. Multiple Thyrotropin Beta-Subunit and Thyrotropin Receptor-Related Genes Arose During Vertebrate Evolution. PloS One (2014) 9(11):e111361. 10.1371/journal.pone.0111361 25386660 PMC4227674

[3] Fleming MS Maugars G Lafont A-G Rancon J Fontaine R Nourizadeh-Lillabadi R. Functional Divergence of Thyrotropin Beta-Subunit Paralogs Gives New Insights Into Salmon Smoltification Metamorphosis. Sci Rep (2019) 9(1):4561. 10.1038/s41598-019-40019-5 30872608 PMC6418267

